# The Use of Eye Gaze Data and Personality Traits: A Scoping Review of the Literature

**DOI:** 10.1002/wcs.70008

**Published:** 2025-06-11

**Authors:** Jan Skala, Kangsoo Kim

**Affiliations:** ^1^ Czech Technical University in Prague Prague Czechia; ^2^ University of Calgary Calgary Alberta Canada

**Keywords:** eye, personality, review, tracking, trait

## Abstract

This scoping review examines the use of eye movement tracking in personality research across various domains, including job interviews, education and training, human‐robot interaction, and user interface design. Eye‐tracking has proven effective in capturing behavioral cues linked to personality traits such as emotional responses, leadership potential, and learning preferences. To map existing research and identify prevailing use case scenarios, a systematic search was conducted in the ACM and IEEE digital libraries. From an initial pool of 170 studies, 21 met the inclusion criteria and were subjected to full‐text analysis. The purpose of this review is to provide a structured overview of current research trends, methodological approaches, and application contexts. Its contribution lies in synthesizing key insights and highlighting opportunities for future research, particularly in the use of eye‐tracking for advancing personalized technologies and behavior‐based analytics in fields such as education, marketing, and psychological analysis.

## Graphical abstract

Illustration of the systematic screening process that distilled an initial pool of 170 studies to 21 key articles, providing a comprehensive overview of eye‐tracking research in personality assessment. This review aims to explore how eye movements can provide valuable insights into underlying personality traits and cognitive processes.

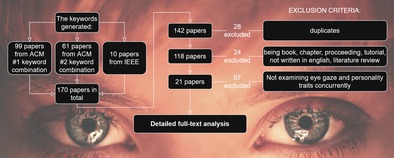

## 1 Introduction

Eye movements and their monitoring: is it a scientific dead end or an exciting domain ready to function as a future telescope into our inner workings? What comprehensive insights can be gathered from our ocular behavior, including the estimation of personality traits, and with what precision? In which spheres of application is the technology currently being tested and examined? Is virtual reality (VR) implicated? To what extent have the outcomes of published experiments proven successful? The objective of this literature review is to provide a comprehensive overview of the use of eye gaze data in the estimation of personality traits, to structure it, and to discuss potential approaches and solutions.

The utilization of eye movement tracking technology is already under experimentation for various purposes, including the study of learning processes, medical diagnostics, and as an input control element (especially for individuals unable to use conventional control peripherals due to disability). Moreover, this technology has found applications in the marketing and advertising sector, primarily for assessing the effectiveness of various marketing strategies and attitudes. Frequently, acute conditions such as stress or emotions are the focus of measurement and examination. However, as it becomes evident (and the results of this survey demonstrate), this field remains relatively new and less explored.

The typical outcomes obtained from eye movement monitoring technology provide details on fixations and saccades. These metrics allow us to understand where a participant looked, how long they focused on a particular area, the sequence in which they observed different points of interest, and what captured their attention the most. Additionally, we can examine patterns of blinking and changes in pupil size.

The new nature of eye movement tracking technology in diverse applications underscores the need for comprehensive exploration. This innovative technology holds promise not only for understanding immediate conditions like stress and emotions but also for exploring territories of learning processes and medical diagnostics. The outcomes of this ongoing inquiry contribute to the evolving understanding of this emerging field.

For the purposes of this review, we explored human attributes that go beyond acute conditions of an individual. While it is established that eye movement tracking can measure immediate aspects like stress levels and concentration, our research focused on the possibility of measuring traits—those either present from birth or subject to prolonged development within an individual. Although the immediate emotional state of the test subject may be indirectly linked to the testing moment, it does not constitute the primary focus of our investigation in this review. A prime candidate for these deeply rooted human attributes, discoverable or not through eye movement tracking (the focal point of this study), is the domain of personality traits. These are a collection of human characteristics shaping behavior, cognition, and emotional patterns, influenced by both environmental and biological factors. Personality traits are systematically classified across various categories and frameworks. Our examination emphasizes the widespread adoption of the Big Five personality trait system.

The Big Five constitutes a personality trait model with five dimensions, encompassing openness, conscientiousness, extraversion, agreeableness, and neuroticism. These traits are commonly referred to by the acronym OCEAN in the specified order. Openness reflects an individual's receptiveness to novel experiences and activities. Conscientiousness characterizes one's level of orderliness, organizational skills, and consideration. Extraversion pertains to the capacity for socialization, emotional expressiveness, and talkativeness. Agreeableness is linked to cooperative tendencies, empathy, and a willingness to assist, reflecting prosocial behavior. Neuroticism is associated with emotional instability, mood fluctuations, and a propensity for anxiety and sadness. Each of these traits can be described along a spectrum, allowing for categorization as high, low, or intermediary, reflecting varying degrees or levels within the context of personality evaluation (Paunonen and Jackson 2000).

Some automatic human reactions to stimuli, like changes in pupil size or sweating, are challenging to regulate. This lack of voluntary control allows us to gather genuine data unaffected by conscious intent. When utilizing such data to assess human characteristics, such as personality traits, we can reasonably assume the authenticity of the results.

In the near future, as Industry 4.0 continues to advance, various departments, including Human Resources (HR), will face increased demands. The term Industry 4.0 refers to the fourth industrial revolution, characterized by the integration of technologies like IoT, AI, robotics, and big data into manufacturing. The HR function, in particular, will encounter increased requirements, necessitating a more thorough extraction of information from candidates. However, the challenge lies in ensuring the authenticity of this information, a task that proves complex when relying on conventional methods such as questionnaires and interviews. The distinctive advantage offered by the utilization of eye movement tracking, alongside other nonverbal and verbal cues, lies in its capacity to remotely estimate personality traits and related characteristics, particularly in the online domain. This technological innovation makes it easier to extract pertinent information from both pre‐recorded videos and live online sessions, covering diverse contexts such as presentations, group events, or online interviews.

The present review study aims to provide a comprehensive overview of the existing literature on the measurement of eye movements within the context of assessing personality traits. This involves an exploration of practical technologies, identification of use case scenarios, and an examination of potential combinations with other multimodal cues. Furthermore, the paper is designed to present and discuss the empirical findings derived from relevant studies, thereby affording the reader insights into the effectiveness of distinct approaches.

The scientific questions for this study are as follows:

- Q1: Can an individual's personality traits be measured through eye movement tracking?
- Q2: In what contexts do the studied studies address the measurement of eye movements in relation to personality traits?
- Q3: Do researchers in the reviewed studies utilize VR technology?

## 2 Methodology

**FIGURE 1:**
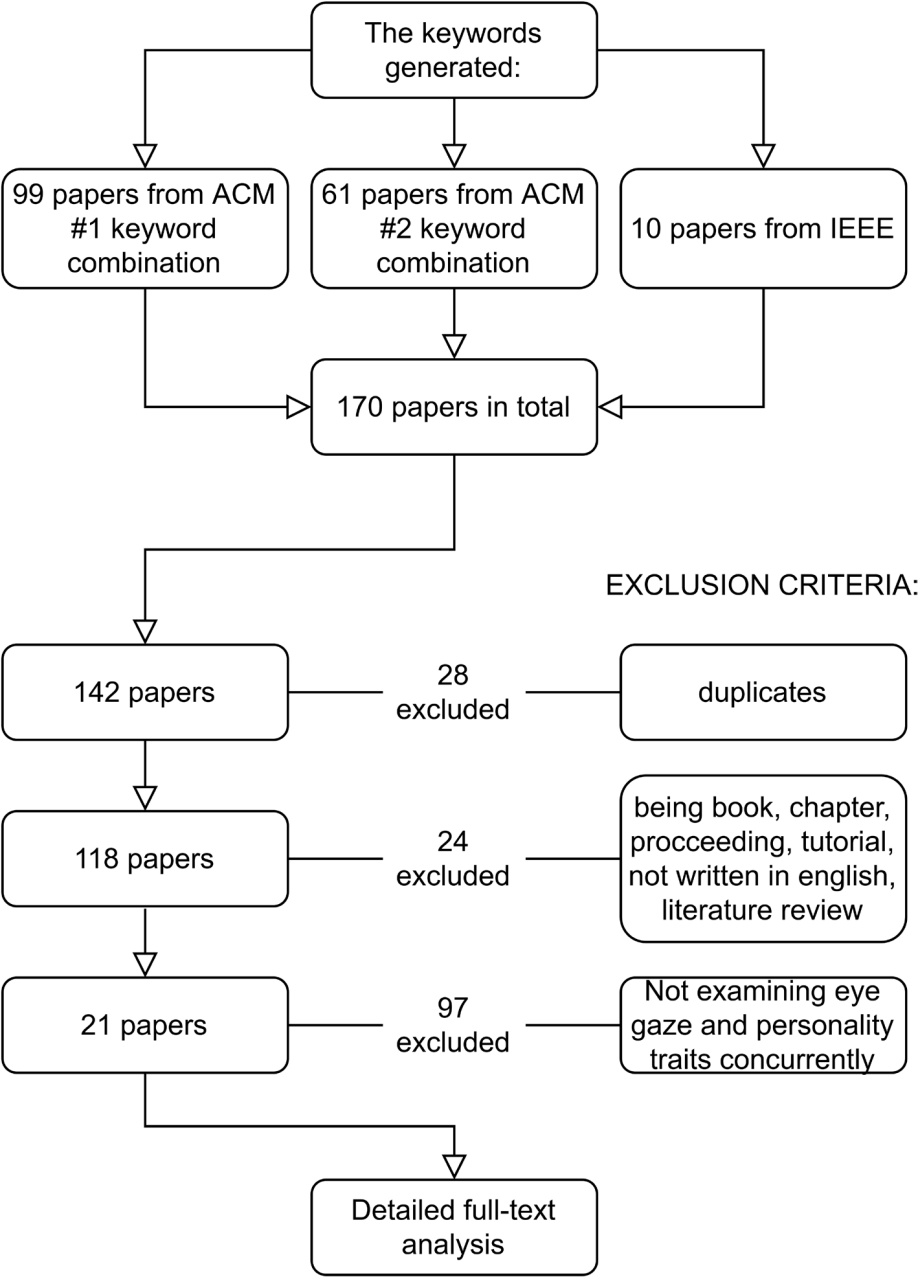
PRISMA scheme.

In this section, the literature search, screening, and categorization procedures follow the methodological framework outlined by Arksey and O'Malley (2005) and Munn et al. (2018). The screening procedure is visually represented in Figure 1 using the PRISMA scheme.

We conducted the literature search using the keywords below on March 10th 2022, in two digital libraries: Association for Computing Machinery (ACM) and Institute of Electrical and Electronics Engineers (IEEE).

On ACM we used two different combinations of keywords:

[[All: eye] OR [All: gaze]] AND [All: “personality trait*”] AND [[All: detect*] OR [All: recogni*] OR [All: sens*] OR [All: perce*] OR [All: track*]] AND [Publication Date: (01/01/2012 TO 03/31/2022)]: 99 results.

[[All: “eye”] OR [All: “gaze”]] AND [All: “personality trait*”] AND [[All: “detect*”] OR [All: “recogni*”] OR [All: “sens*”]] AND [Publication Date: (01/01/2011 TO 03/31/2022)]: 61 results.

On IEEE those keywords were used:

(“All Metadata”: “eye” OR “All Metadata”: “gaze”) AND (“All Metadata”: “personality”) AND (“All Metadata”: detect OR “All Metadata”: recognize OR “All Metadata”: recognize OR “All Metadata”: sense): 10 results.

A time constraint from January 2011 to March 2022 was set during the search. The reason for selecting this timeframe was the increased awareness of this technology during that period, leading to the subsequent publication of articles. The search result is composed of 10 papers from IEEE, 160 papers from ACM. In total, 170 papers were forwarded to the screening stage.

To filter out papers that are not qualified or relevant to the topic of this literature review, the following exclusion criteria were applied during the screening stage: does not deal with the issue of eye gaze; does not deal with the issue of personality traits; being a book; being a book chapter; being a tutorial; being a proceedings conference paper; being a literature review paper; not written in English.

These specific exclusion criteria were chosen based on a thorough examination of individual items found using the mentioned keywords. Books and their chapters, tutorials, and conference papers in proceedings were considered too vague and irrelevant. No literature review paper on this topic was found (emphasizing the importance of generating a literature review on this subject). Materials written in languages other than English were excluded due to difficulties in accessibility and understanding. The authors of this review aimed to ensure that the cited literature would be easily accessible and understandable to as diverse an audience as possible. The last‐mentioned criteria did not need to be applied (given the use of English keywords); nevertheless, the authors considered it important to incorporate them into the filtering process to maintain maximum transparency of the sorting procedure.

Many studies that have been excluded are highly intriguing and scientifically valuable, but they were excluded precisely because they do not address the issues of eye gaze or personality traits (Bhattacharya et al. 2018; Chidambaram et al. 2012; Ishii et al. 2020; Iwasaki et al. 2019; Jraidi et al. 2013; Kujala et al. 2016; Lallé et al. 2015; Lin and Lee 2018; Ostberg et al. 2016; Paradeda et al. 2019, 2020; Rayon et al. 2016; Segalin et al. 2017; Takayama et al. 2021; Toker et al. 2014; Wache et al. 2015; Wang et al. 2015).

Given the above criteria, we conducted four rounds of screening: merged duplicates and checked the title and abstract of the papers for relevance in the first and second rounds of screening; conducted the third round of relevance checking on full texts, and the fourth round of explicit tagging. The full text of a paper was initially checked by the first author. Ambiguous papers were discussed among the authors and reviewed with consensus. The tagging categories were agreed upon by the authors and are introduced in the following section. After screening, 149 papers were excluded. The remaining 21 papers were used for our detailed full‐text analysis.

To gain a better understanding of each study's core ideas, we had to organize the papers using different criteria. These criteria were carefully chosen to directly impact the subjects discussed in our literature review. Here are the specific criteria we focused on:

- What prompted the utilization of eye tracking, and what specific outcomes were sought?
- What eye tracking equipment was employed for the study?
- Were personality characteristics considered as input or output factors?
- Did the study involve the presence of a robot or an agent?
- Were additional sensors employed in the research?
- Was supplementary verbal or nonverbal data incorporated?
- What specific use case scenarios were examined?
- What was the research type (e.g., user study, method…)?
- How many participants actively participated in the study?

Following the processing of this data and the evaluation of individual studies against these criteria (documented in a structured spreadsheet), we distilled our observations into specific conclusions. To ensure readability and provide a comprehensive overview of the studies on this topic, the main section of the article—particularly the literature reviews—was meticulously structured using these selected criteria. This approach aimed to enhance clarity and illuminate the primary themes explored within the research.

## 3 Literature Reviews

**FIGURE 2:**
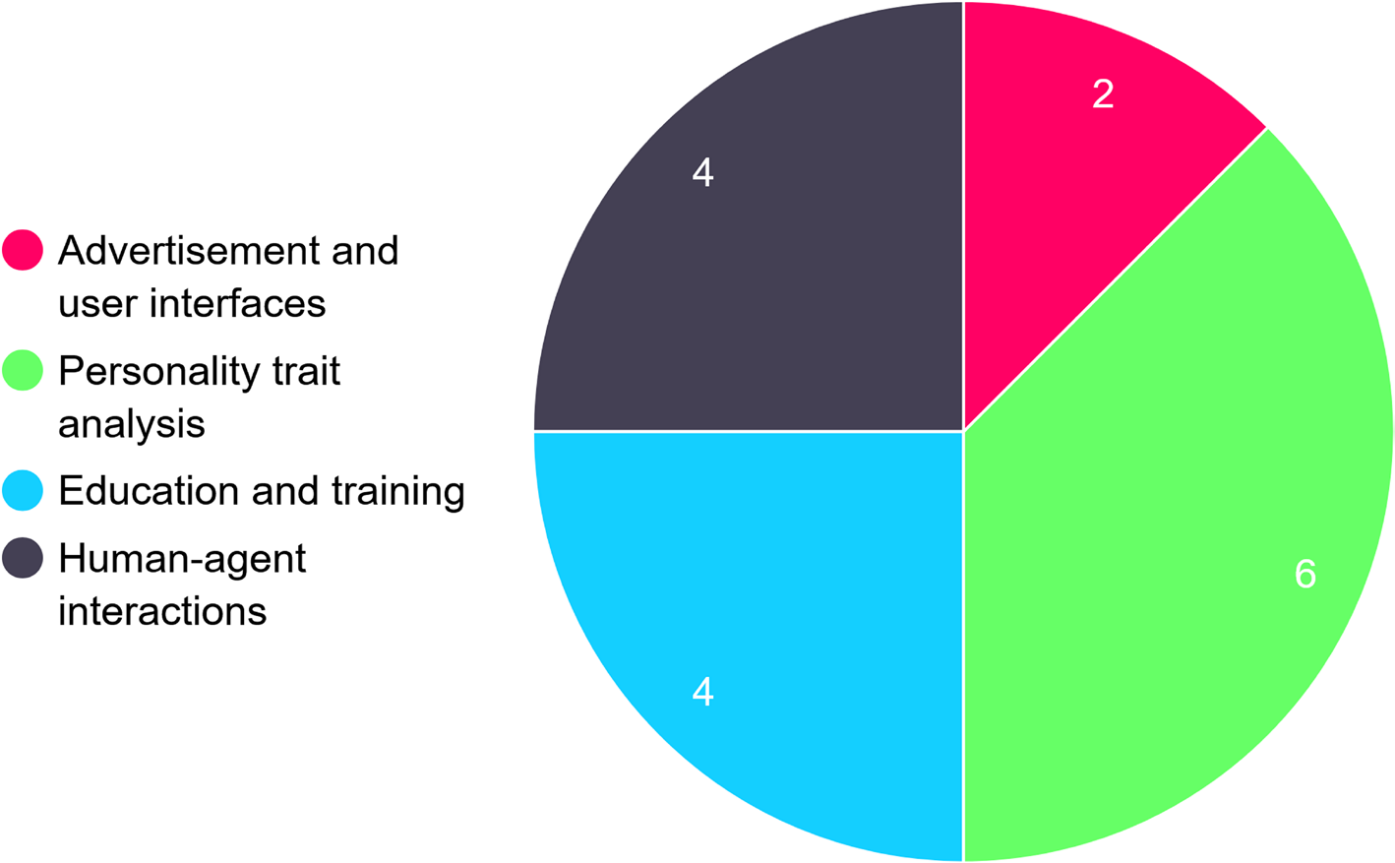
Distribution of eye tracking applications across various fields in 21 studies.

**FIGURE 3:**
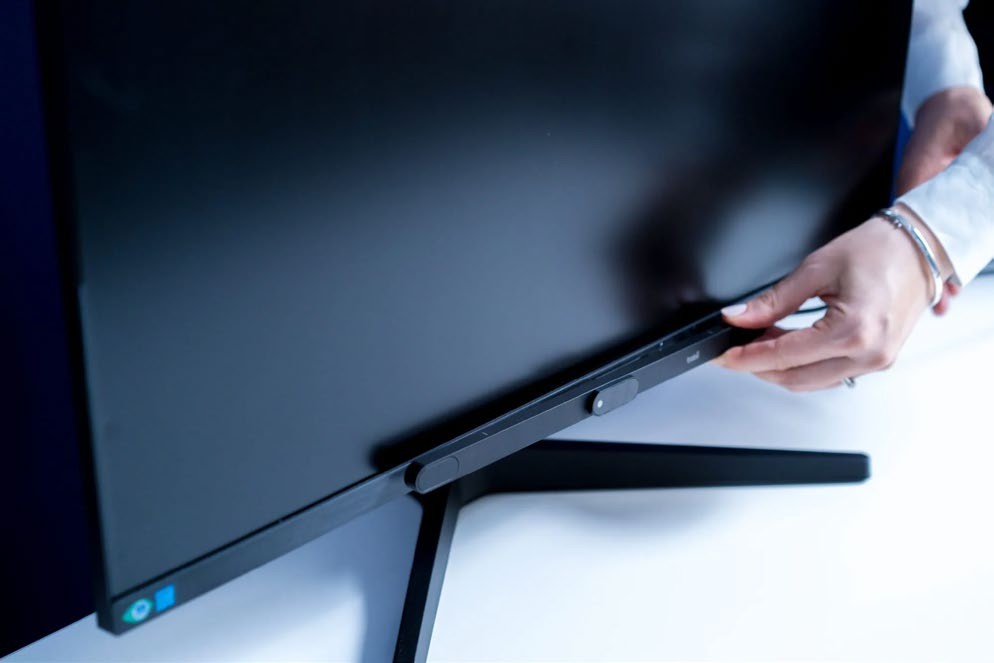
The screen mount of a screen‐based eye tracker (Miseviciute n.d.).

**FIGURE 4:**
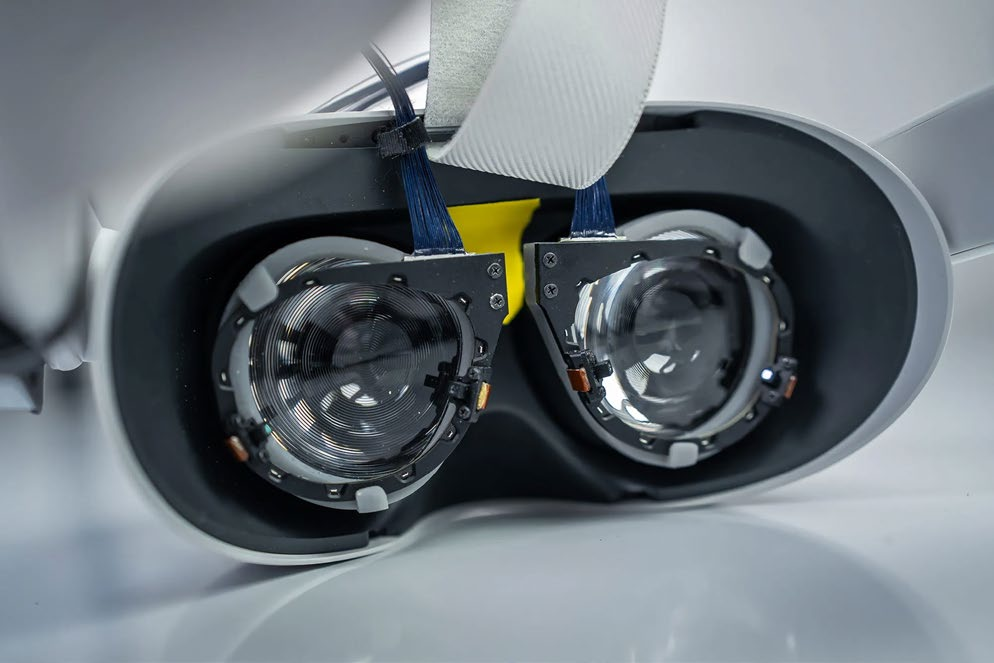
VR headset with Tobii eye tracking technology (Miseviciute n.d.).

In this section, we present in‐depth qualitative reviews of the selected papers based on the classification categories described in Section 2: use contexts, other multimodal cues, and eye tracking devices. Refer to Figures 2, 3, 4 for a clear overview of the distribution of use case scenarios across the analyzed studies.

### 3.1 Contexts of Use

#### 3.1.1 Job Interviews

Five research papers were dedicated to examining the utilization of eye gaze and personality traits within the context of job interview settings as a case study.

Batrinca et al. (2011) presented an automatic method to detect user's personality traits during a short period of self‐presentation (30–120 s). A total of 89 participants were recruited to evaluate the proposed personality detection method, and conscientiousness, emotional stability, and extraversion of the participants were measured. The findings indicated that the suggested methodology demonstrated a proficiency exceeding 70% in predicting some personality traits. Furthermore, the results revealed a tendency for individuals to exhibit heightened conscientiousness with advancing age. Additionally, the study showed a pattern where men displayed greater emotional stability compared to women, while women exhibited a tendency toward greater extraversion.

Dissanayake et al. (2021) accomplished the evaluation of personality traits within the context of online interviews. In their study, they developed a methodological approach to examine the personality of job candidates through the analysis of recorded nonverbal behaviors, providing a foundation for fair hiring determinations. The findings indicated that examined personality traits could be identified with a precision surpassing 75%, using cues derived from nonverbal behaviors, notably those associated with eye gaze.

Chen et al. (2016) introduced an innovative automated data processing methodology using visual behavioral data, such as eye movements and facial expressions, in conjunction with other verbal cues. This approach holds promise for predicting the personality traits of interviewees, thereby facilitating informed hiring decisions. A total of 36 participants engaged in the research, with the automated assessment providing insights into the interviewees' social skills across four dimensions: communication skills, interpersonal skills, leadership, and persuasion and negotiation. The authors introduced a new multimodal corpus to support the application of Social Signal Processing (SSP) and utilized Behaviorally Anchored Rating Scales (BARS) in the context of multimodal research. Importantly, they emphasized the use of the Doc2Vec method, a vector representation technique that assigns vectors not only to words but also to their meanings. This nuanced approach enhances the accuracy of predictions, as it allows the computer program to grasp the semantic nuances present in word combinations—and thereby, to better comprehend what the participant is expressing.

Okada et al. (2015, 2019) directed their attention toward the analysis of multi‐party behavior to assess personality traits, emergent leadership skills, and communicative competence within an interview setting for the purpose of informing hiring decisions. The study involved the simultaneous examination of multiple human subjects (co‐occurrence), where, for instance, one member spoke while others displayed non‐verbal cues such as nodding. The study's findings revealed that classifiers trained with co‐occurrence features significantly enhanced accuracy, demonstrating an improvement ranging from 3% to 13%. Specifically, the accuracy rates achieved were 70% for extraversion, 67% for agreeableness, and 66% for openness to experience.

#### 3.1.2 Education and Training

Four scholarly articles were dedicated to the exploration of education and training domains. Specifically, Fekry et al. (2019) conducted an analysis of videos featuring student group presentations, aiming to establish a correlation between student presentation behavior and two models: VARK (Visual, Aural, Read/Write, Kinaesthetic) and PAEI (Producer, Administrator, Entrepreneur, Integrator). These models describe distinct areas of individual proficiency. The outcomes of the study revealed that individuals categorized as “Administrators” and “Kinaesthetics” exhibited longer presentations, whereas those classified as “Integrators” and “Aurals” demonstrated shorter presentations. Furthermore, individuals identified as “Producers” and “Visuals” displayed a pronounced focus on eye movements, while “Administrators” and “Visuals” were the loudest.

Sun et al. (2017) tried to enhance the interface of the Chinese online study system, specifically the Learning Analytics Dashboard (LAD), with the aim of personalization. The goal was to examine the learning methodologies employed by each student and identify any potential correlations with their personality traits. Fifteen specific behavioral indicators were extracted from a group of 662 high school students. The findings of the study revealed a noticeable correlation between personality traits and learning behaviors. Particularly, ocular measurements showed varying levels of interest among students in various components of the LAD. Learning advice, comparative analysis of quiz scores within the class, engagement in learning scenarios, and participation in forums attracted the most attention. However, the research team faced challenges in establishing a conclusive link between ocular activity (points of interest in the LAD) and specific personality traits.

Carenini et al. (2014) focused on optimizing the efficiency of gathering information from visualizations, specifically bar graphs. This study aims to investigate how graphs can be tailored to individual users through interventions, such as selectively highlighting specific elements within the graph. To assess the effectiveness of these interventions, a user study was conducted with a participant pool comprising 62 individuals. To identify potential correlations, the study examined three cognitive abilities of users: perceptual speed, verbal and visual working memory, and the Locus of Control personality trait. The findings of the study indicated that interventions involving highlighting significantly enhance the processing of visualizations, resulting in improved task performance and increased perceived usefulness among users. Furthermore, the study revealed that the quality of individuals' cognitive abilities correlates with their performance in complex tasks. However, no noticeable connection emerged between the study outcomes and the personality trait known as Locus of Control.

Chollet et al. (2018) conducted an investigation into public speech training, with a particular emphasis on examining the relationship between human characteristics and the performance of public speaking. Initially, the speaker's behavioral data were systematically collected, processed, and afterward evaluated. Throughout this process, a virtual audience was incorporated, expressing varying levels of arousal and valence through non‐verbal behaviors such as head nods, postures, and gaze patterns. The study encompassed a participant pool of 35 individuals. The study outcomes revealed that neuroticism, extraversion, and conscientiousness have a significant impact on the efficacy of public speaking. Predictably, the speaker's Public Speaking Anxiety score was identified as having an adverse effect on their performance.

#### 3.1.3 Human‐Agent Interactions

Four research papers were undertaken to scrutinize user personality traits within the domain of human‐robot interactions or to generate nonverbal behaviors of a virtual agent based on personality traits. In the realm of human‐robot interactions, Shen et al. (2020) introduced a computational framework designed to enable a robot to identify personality traits based on the nonverbal communication cues exhibited by the user. The experimental setup involved the robot named Pepper posing questions to individual participants (15 in total), who responded in a natural manner. Simultaneously, the robot captured nonverbal features from each participant's habitual behavior utilizing its on‐board sensors and first‐person perspective. The accuracy rates for personality trait identification were as follows: Extroversion at 64%, Agreeableness at 89%, Conscientiousness at 87%, Emotional stability at 76%, and Openness at 72%.

Abe et al. (2017) introduced a methodology aimed at enabling robots engaged with children to deduce essential personality traits, specifically extraversion and agreeableness. The experimental group consisted of 29 children, each engaging with the tele‐operated robot for a duration of 30 min, with personality trait estimation conducted during the initial 10 min of interaction. Following the experiment's conclusion and the application of SVM machine learning for data analysis, the authors concluded that the robot demonstrated an ability to estimate extraversion with an accuracy of 71% and agreeableness with an accuracy of 60%.

Aly and Tapus (2013) emphasized the importance of aligning a robot's communication with an individual's personality traits while also expressing its own personality traits. Human speech underwent analysis, and subsequent personality trait estimations guided the robot's adaptive behavior. The experiment involved 21 participants. The study confirmed the hypothesis that an extroverted person exhibits a preference for interacting with an extroverted robot, whereas an introverted individual tends to favor interaction with an introverted robot. Additionally, the hypothesis that a robot employing both gestures and speech in communication receives greater engagement and is perceived as more fitting and natural compared to a robot relying solely on speech was supported by participant responses.

In the development of realistic interactive virtual agents, Ruhland et al. (2015) attempted to determine the potential identification of specific personality traits solely through an avatar's eye gaze, blinks, and head movements, without additional facial animation cues. Using facial capture technology, human facial expressions were recorded and then applied to virtual avatars. The study, involving 24 participants, aimed to evaluate the impact of these avatars on individuals. The findings of the study affirmed the feasibility of estimating specific personality traits exclusively from eye and head movements, with no noticeable difference in perception between realistic and cartoon avatars. Furthermore, the study revealed no significant difference in appeal between the realistic and cartoon models.

#### 3.1.4 Personality Trait Analysis

Within the reviewed literature, six papers were identified with a primary focus on personality traits themselves. The primary objective of these papers was the estimation of personality traits and their contextualization within the scope of the respective studies.

Berkovsky et al. (2019) and Taib et al. (2020) employed eye‐tracking technology and skin conductivity measurements to estimate personality traits, specifically the Dark Triad, BIS/BAS, and HEXACO. In addition, Berkovsky et al. included the LSRP, NPI‐16, and MACH‐IV questionnaires as additional measures. The rationale behind selecting these measures was their lack of conscious control by the subjects. The study involved 21 participants, and responses were triggered by external emotional stimuli, subsequently captured and assessed through machine learning methods. Notably, eye tracking provided more precise results compared to skin conductivity, and dynamic video stimuli surpassed static stimuli. The optimal results were achieved through combinations, with an accuracy rate of approximately 90% in determining personality traits.

Gehrer et al. (2018) aimed to measure differences in eye gaze behavior during facial perception between antisocial male offenders and healthy individuals. Psychopathic personality traits and aggressive behavior were evaluated through questionnaires and interviews, specifically using the SRP‐III and BPAQ scales. The research included 21 male offenders and 21 healthy individuals. Static images of faces displaying various emotional expressions served as stimuli, and participants were tasked with categorizing the presented emotional faces. However, no noticeable differences in terms of eye gaze behavior between the groups were identified.

Chávez‐Martínez et al. (2015) attempted to extract and predict personality traits and emotions from internet video blogs. The Big Five model was employed as the underlying personality framework. The researchers opted for a multi‐label approach, recognizing natural correlations between emotions and personality traits. YouTube video blogs served as the datasets, and various machine learning methods were applied for information processing. The findings indicated the possibility of predicting personality traits and emotions from videos, highlighting existing correlations between emotions and traits. Notably, the utilization of the multi‐label method contributed to an enhanced accuracy of the results.

Thomas et al. (2019) conducted an analysis of video reviews of books with a focus on multimodal analysis. The primary objective was to identify differences in the geographical context and the method of automatic recognition, specifically between the Western and non‐Western communities (India). The dataset comprised 4478 videos from 21 Indian and 34 Western channels, all presented in English. The study revealed the ability to automatically differentiate between Indian and Western authors, with verbal content proving more relevant than audio‐visual nonverbal cues. Recognition accuracy surpassed 90% for audio, visual, and linguistic features, reaching 98% accuracy when combining all three features.

Zhang et al. (2019) aimed to investigate group behavior by introducing an algorithm estimating the Visual Focus Of Attention (VFOA), coupled with prosodic voice analysis. The BFI‐10 and EI scales were utilized to assess personality traits and emotional intelligence, respectively. The researchers considered facial behaviors, eye gaze direction, tone of voice, and the level of engagement, linking this data to personality traits (Big Five) and leadership skills. The study involved 45 participants in 14 group meetings. The research demonstrated the VFOA system's ability to determine the focus of attention with 90% accuracy. Moreover, the combination of VFOA and prosodic data enabled the prediction of emergent group leaders with 64% accuracy and dominant contributors with 86% accuracy. A correlation was observed between personality traits and group behavior.

#### 3.1.5 Advertisement and User Interfaces

Two papers fall within this specific domain of utilizing eye gaze data. Evangelou and Xenos (2020) investigated the effectiveness of static banner advertising, seeking to understand individuals' perceptions of these banners and the factors influencing their performance. The research employed interview, questionnaire, and eye tracking data as analytical tools, examining the relationship between advertising perception and personality traits. The experiment involved 18 participants, utilizing a simulated online store with advertisements created for the study's purposes. The confirmed existence of “banner blindness,” a phenomenon where individuals do not perceive advertising, was noted. Only three out of the 18 “customers” were affected by the banners, and a connection between neuroticism and the disregard for advertising was identified.

Lallé and Conati (2019) explored the potentials of customization and adaptation within the User Environment, concentrating on the MetroQuest application designed for public engagement in urban planning. This paper investigates how users tailor the User Interface (UI) in relation to their characteristics, encompassing Perceptual Speed, Visual Working Memory, Visualization Literacy, and the Locus of Control personality trait. A user study, attended by 46 participants, was organized by the authors. The study's outcomes revealed that 33% of users modified their UI, and the effectiveness of these adjustments was influenced by their visualization literacy and Locus of Control. Users with higher visual literacy engaged in more visual comparisons than those with lower visual literacy.

### 3.2 Other Multimodal Cues

All the reviewed studies use the eye gaze as a source of valuable information on which the calculations and conclusions are based. In most cases, eye behavior is very important or the primary cue. In the vast majority of cases, however, this is combined with other human features to create multimodal monitoring/measurements.

These other cues reported in the reviewed papers include: head movement, body movement, hand gestures, facial expressions, nonverbal voice characteristics, verbal (linguistic) characteristics, pupil size, GSR (skin conductance) and distance between person and scanning device.

In addition to the eye gaze, the non‐verbal voice feature was the most commonly used feature (12 studies); the second most commonly used were facial expressions (8 studies) and the third was head movement (7 studies).

The biggest number of features in their research has Thomas et al. (2019). His team used eye gaze data, head movement, facial expressions, and nonverbal and verbal voice characteristics.

#### 3.2.1 Body Gestures and Facial Expressions

Combining eye gaze with other body movements was very common in the peer‐reviewed studies. According to the results of the reviewed research, the multimodal approach increases the accuracy of estimates of human characteristics. In addition, if the test subject is captured by a camera, it is offered to extract other movement features than just eye gaze.

Body gestures are important for recognizing personality traits, as Aly and Tapus (2013) state in their study. In this case, the robot recognizes the personality traits of the person and tries to behave in the same way. The robot uses gesticulation for this purpose. The results of the study showed that thanks to the gestures, the participants were able to better distinguish what personality traits the robot mimicked.

Eight studies applied head movements, seven studies body movements, and eight studies facial expressions. When using cameras to measure eye gaze, head movements and facial expressions were usually extracted simultaneously with eye gaze.

For example, Okada et al. (2019) addressed group behavior in their study. In addition to the eye gaze of the individual participants, their movement was also monitored—all participants at the same time. The researchers measured whether the participants were moving the body or the head (e.g., nodding).

Chávez‐Martínez et al. (2015) followed body movements, head movements, and facial expressions when analyzing videos from the Internet. From the facial expressions, they successfully calculated the valence and emotions of the given participant.

#### 3.2.2 Linguistic and Vocal Cues

A lot of papers employed voice features as well. These vocal features can be divided into two groups: nonverbal and verbal. The analysis of nonverbal features was used in 12 studies and verbal in 4 studies.

In the case of nonverbal vocal characteristics, it is a prosodic analysis of the voice. Very often it is about pitch and acoustic energy. For example, Chen et al. (2016) in their research on robot‐human interaction used voice pitch, voice energy, and mel‐frequency cepstral coefficient (MFCC) analysis. Thanks to this, the robot was able to increase the accuracy of estimating the personality traits of the person with whom it communicated. The extracted nonverbal features were very similar in all cases used.

In the case of verbal analysis, it involves extraction of the spoken word, machine translation into text, and linguistic analysis. For example, in their study, Thomas et al. (2019) conducted a linguistic analysis of internet content creators for the purpose of recognizing geographical differences (in this case, Indian authors vs. Western authors). The specific tools used were Count vectorizer, tf‐idf, and Lexical features (use of words and vocabulary) analysis using the Linguistic Inquiry and Word Count (LIWC). Chen et al. (2016) extracted and analyzed pronunciation, grammar, and vocabulary use for the purpose of estimating personality traits.

#### 3.2.3 Physiological Cues

Two studies have gone the way of monitoring physiological features. Berkovsky et al. (2019) and Taib et al. (2020) have resorted to tracking features that are uncontrollable by will, and the results will therefore be legitimate. The first physiological feature was pupil dilatation. Using SMI eye‐tracking glasses, they measured vertical and horizontal pupil dilatation, a feature that may be related to personality traits or fear and anxiety. The second physiological feature used in the study was Galvanic Skin Response (GSR), measured with Procomp Infiniti sensor. It is a measurement of skin conductivity caused by perspiration, which reflects autonomic nervous activity elicited by physiological responses.

### 3.3 Eye Tracking Devices

Different studies have approached the way in which eye movements are sensed differently. There were two main approaches: the use of dedicated eye tracking hardware or the use of an ordinary camera. In the case of using the camera (like a webcam, for example), eye movements were estimated by special software. For instance, Zhang et al. (2019) or Thomas et al. (2019) used OpenFace software. It is a modern face recognition system that uses neural networks and deep learning. This program estimates eye gaze, for example, from the direction of head rotation. Other studies using a conventional camera have addressed this in the same way, or very similarly. The great advantage of this solution is that it is possible to analyze already recorded videos from the Internet (e.g., from social networks), or that the analysis of personality can take place remotely (e.g., during an online interview). Therefore, there is no need for a laboratory environment and the measurement itself is not obstructive. Disadvantages include low measurement accuracy. In the examined papers, this technology was used to measure approximate directions of gaze (e.g., to calculate who the person is looking at in a multi‐person meeting, or whether the person is looking at the screen or not).

A far more accurate technology is the use of dedicated eye tracking hardware. These devices track eye movements with high accuracy and can be used to track exact points of interest on the screen. From the output of these devices, it is possible to monitor the saccades, fixations, blinking, and pupil size. For example, heatmaps and other useful analyses can be created from this data, which describe the eye behavior of the participants in detail. Gehrer et al. (2018) used the SR Research EyeLink 1000 camera to accurately monitor the response of healthy individuals and violent offenders to human faces and emotions. For example, they watched what parts of the face these people focus on when reading emotions. Sun et al. (2017) used the Tobii X2‐60 camera to track what information students focused on within the UI application, which contains overview data and statistics about their studies. Berkovsky et al. (2019) and Taib et al. (2020) used their SMI Eye‐Tracking Glasses study, which may be less intrusive than eye tracking cameras. Various stimuli (static and video) were displayed on the screen, and the researchers used these eye tracking glasses to successfully monitor the participants' eye behavior. Carenini et al. (2014) and Lallé and Conati (2019) used the Tobii T120, a computer monitor with a built‐in eye tracking camera. Carenini et al. (2014) used the device to monitor ocular behavior in information processing (specifically bar graphs). Lallé and Conati (2019) used this device to explore the effectiveness of the UI and the ways in which people customize their UI.

## 4 Discussion

In the realm of the explored scientific studies, it becomes clear that the current state of knowledge regarding the use of eye behavior monitoring in understanding personality traits has advanced into a stage marked by successful experiments and the ongoing development of new technologies. These emerging technologies hold great promise, expected to make significant contributions across various areas such as psychology, psychiatry, human resources, education and training, human–robot interaction, user environment optimization, and marketing.

The findings from a thorough examination of these studies confirm the ability to estimate personality traits by carefully looking at eye gaze behavior. Moreover, a noticeable improvement in the accuracy of these estimations is observed when additional features are thoughtfully included in the calculation process. These extra features include a range from facial expressions and subtle head and body movements to a detailed analysis of nonverbal vocal characteristics and the intricate details of linguistic elements. This comprehensive approach not only boosts the precision of personality trait estimation but also underscores the potential for a thorough understanding of individual characteristics by considering a variety of behavioral cues.

An interesting point to note is that, despite exploring different scenarios, all of these studies ultimately aimed at measuring eye movements in relation to personality traits. While some focused on these traits regardless of specific situations, this recurring theme consistently appeared across a wide range of investigations. It underscores a notable pattern across these studies, underscoring the significance of exploring personality through the lens of eye movements.

In the comprehensive exploration of various studies, researchers frequently opted for the use of traditional fixed cameras paired with specialized software to precisely track eye movements. The primary focus was on estimating the direction of gaze, with an emphasis on identifying subtle details such as the person in the room at whom the subject was looking, the duration of the gaze, and the sequence of alternation between different individuals. It's worth noting that this approach, though somewhat constrained in terms of absolute accuracy and sensitivity to factors like lighting conditions and camera quality, compensates by not intrusively affecting participants' movements or overall comfort during testing.

In scenarios where precise eye movement tracing was essential, researchers turned to dedicated hardware solutions, such as specialized cameras or glasses. However, the adoption of such tools introduces considerations and limitations, both within the controlled experimental environment and in potential real‐world applications. High‐precision cameras tailored for monitoring eye movements, for instance, necessitate a fixed head position, inevitably imposing constraints on the comfort level of test subjects. Furthermore, this method inherently calls for meticulous calibration procedures, demands a high level of expertise from laboratory technicians, and underscores the importance of carefully configuring the overall laboratory or experimental setting to ensure optimal performance.

A significant consideration in some studies is that people might act differently in a controlled lab setting compared to their usual surroundings. This difference can notably affect the precise measurement of personality traits, especially those related to extraversion. A relatable scenario involves interactions with humanoid robots—creations designed with human‐like proportions but often characterized by somewhat exaggerated and humorous movements. In such situations, maintaining natural behavior becomes challenging. The person being tested may unconsciously grapple with uncertainty, pondering whether they are interacting with the robot as they would with a person or treating it more like a machine. This inherent uncertainty can manifest in the emotions expressed during the interaction, impacting how the robot should read and evaluate them.

Interestingly, none of the examined articles incorporated virtual reality (VR) technology, despite the feasibility of integrating precise eye movement monitoring devices into VR headsets. Enhancing the immersive aspect could yield intriguing outcomes, especially in studies focused on public speaking training or research emphasizing the significance of external stimuli. However, it is apparent that the undeniable advantages associated with virtual reality are counterbalanced by the drawbacks outlined in the preceding paragraph. These include the need for a detailed setup of experiments involving motion cameras, calibration requirements, and other complexities encountered in practical applications. Additionally, related issues such as problems with balance or feelings of nausea are considerations in the VR context. It is important to mention the limitations of this scoping review. The limitations of this review arise from utilizing only two databases (ACM DL and IEEE Xplore) and employing a restricted set of keywords and combinations. Nevertheless, it is crucial to acknowledge that numerous keywords and their diverse combinations were explored, though yielding minimal or irrelevant results in most cases. Although the number of keyword combinations used may seem limited, this selection was the result of extensive testing and refinement, ensuring its effectiveness.

The answers to the scientific questions for this study are as follows:

- Q1: Can an individual's personality traits be measured through eye movement tracking?

Several studies have demonstrated that personality traits can be estimated using eye movement data, although the accuracy and reliability of these assessments vary depending on the technology and computational algorithms applied (Abe et al. 2017; Batrinca et al. 2011; Berkovsky et al. 2019; Dissanayake et al. 2021; Chávez‐Martínez et al. 2015; Chen et al. 2016; Okada et al. 2015, 2019; Shen et al. 2020; Taib et al. 2020).

- Q2: In what contexts do the studied studies address the measurement of eye movements in relation to personality traits?

The reviewed studies cover a range of situations, including Personality Traits Analysis (Berkovsky et al. 2019; Gehrer et al. 2018; Chávez‐Martínez et al. 2015; Taib et al. 2020; Thomas et al. 2019; Zhang et al. 2019), Job Interviews (Batrinca et al. 2011; Dissanayake et al. 2021; Chen et al. 2016; Okada et al. 2015, 2019), Education and Training (Carenini et al. 2014; Fekry et al. 2019; Chollet et al. 2018; Sun et al. 2017), Human‐Agent Interaction (Abe et al. 2017; Aly and Tapus 2013; Ruhland et al. 2015; Shen et al. 2020), Advertising, and User Interfaces (Evangelou and Xenos 2020; Lallé and Conati 2019).

- Q3: Do researchers in the reviewed studies utilize VR technology?

Surprisingly, none of the studies incorporated virtual reality (VR) technology, even though VR headsets with built‐in eye‐tracking technology are available. This observation points to a notable research gap in this area.

## 5 Conclusions

In this thorough literature survey, our focus delved into the complex realm of monitoring eye movements in relation to personality traits. To broaden our scope, we carefully selected studies that used eye gaze measurements while also exploring personality traits, recognizing the potential indirectness of this connection.

Our search covered two prominent libraries, namely ACM Digital Library and IEEE Xplore. After carefully selecting keywords and merging results, an initial pool of 170 studies emerged. Subsequent application of screening criteria led to the exclusion of irrelevant papers, leaving us with 21 articles for a detailed and careful review.

Upon in‐depth examination and contextualization of the gathered papers, a compelling revelation emerged: personality traits can be accurately understood through eye gaze measurements. Leveraging advanced techniques like machine learning and neural networks, these traits can be automatically estimated. Augmenting this approach with additional multimodal data—from body and head movements to facial expressions, nonverbal or verbal voice characteristics, and physiological conditions—further enhances accuracy. Importantly, these features can be extracted in various settings, be it within a controlled laboratory environment or from pre‐existing data sources, such as social media videos.

The surveyed articles presented a diverse array of use case scenarios, extending beyond Personality Traits Analysis to encompass realms like Job Interviews, Education and Training, Human‐Agent Interaction, Advertising, and User Interfaces.

The experiments detailed in these research papers yielded resounding success, affirming a robust link between eye behavior, alongside other mentioned features, and personality traits. This correlation extended to broader human characteristics such as emotions, leadership abilities, or preferences in learning methods, showcasing the multifaceted potential of eye movement analysis in understanding the intricacies of human behavior.

An important finding emerges from the broader discussion: there is a notable lack of research on psychological measurements using eye movement tracking. This gap is particularly striking given the potential of this method, especially in the context of the evolving demands of Industry 4.0 and the changing needs of the workforce. This highlights the method's potential significance in future research and raises questions about the areas that require further exploration and development.

In conclusion, this review reveals the relationship between the technological potential and the limitations of eye‐tracking as a tool for psychological measurement. The absence of extensive research in this area, especially in light of emerging workforce needs, underscores the need for further investigation. The potential of eye movement tracking as a method for understanding human cognition warrants more focused research to explore its application and relevance in contemporary psychological studies.

## Author Contributions

**Jan Skala:** conceptualization (lead), data curation (equal), methodology (equal), writing – original draft (lead), writing – review and editing (equal). **Kangsoo Kim:** data curation (equal), methodology (equal), writing – review and editing (equal).

## Conflicts of Interest

The authors declare no Conflicts of Interest.

## Related WIREs Articles

What eye movements can tell us about sentence comprehension

Expertise differences in cognitive interpreting: A meta‐analysis of eye tracking studies across four decades

## Data Availability

Data sharing is not applicable to this article as no new data were created or analyzed in this study.
